# Retraction: Green synthesis of SiO_2_ nanoparticles from Rhus coriaria L. extract: Comparison with chemically synthesized SiO_2_ nanoparticles

**DOI:** 10.1371/journal.pone.0346847

**Published:** 2026-04-09

**Authors:** The PLOS One Editors

Following the publication of this article [1], concerns were raised about Figs 6 and 7, as well as about the availability of the underlying data. Specifically:

- Fig 6A appears highly similar to the right panel of Fig 4 in [2], which was published in 2017 by different authors.
- There is a mismatch between the reported electron microscope in the Methods sections (Quanta 450) and the banners in Fig 6 (MIRA3 TESCAN).
- In Fig 7B, two regions with a highly similar pixelation pattern were noted.
- The underlying data supporting the study results reported in [1] are not available. At the time of submission, *PLOS One* policy required authors to make the underlying data available with the article.

The comments and data provided by the authors in response to the above-mentioned issues did not resolve the concerns about the published results.

In light of the above unresolved concerns that question the reliability and integrity of these results, the *PLOS One* Editors retract this article [1].

ASM did not agree with the retraction. CYR, AAB, and SMH either did not respond directly or could not be reached.
